# Supplementary material for: Genome-wide association study identifies three PNPLA3/SAMM50 SNPs associated with HCC development in non-viral liver disease
Source: JHEP Rep. 2025 Nov 11;8(2):101673. doi: 10.1016/j.jhepr.2025.101673 (PMC12857342; doi:10.1016/j.jhepr.2025.101673)
Supplement: Multimedia component 1 [file mmc1.pdf]

Genome-wide association study identifies three PNPLA3/SAMM50 SNPs associated with HCC development in non-viral liver disease

Xia-Rong Liu, Tsai-Hsuan Yang, Tung-Hung Su, Szu-Ching Yin, Yi-Ting Chen, Fen-Fang Chen, See-Tong Pang, Ming-Chih Hou, Yen-Chun Peng, Shun-Fa Yang, Peng-Ju Huang, Sing-Lian Lee, Ming Chen, Chih-Yang Huang, Ya-Hsuan Chang, Hsuan-Yu Chen, Hwai-I Yang, Ming-Lung Yu, Chien-Jen Chen, Jia-Horng Kao, Mei-Hsuan Lee

Table of contents

Table S1……..2

Table S2……..4

Table S3……..5

Table S4……..6

Table S5……..7

Table S6……..8

Table S7……..9

Table S8……10

Table S1. Baseline characteristics of the study population in the GWAS phase.

Baseline characteristics	**HCC cases** (n = 765) n	%	**non-HCC controls** (n = 9949) n	%	P-value
**Sex**					<.0001
Male	606	79.22	2827	28.41	
Female	159	20.78	7122	71.59	
**Age** (years)					<.0001
30-39	22	2.88	565	5.68	
40-49	49	6.41	1662	16.71	
50-59	152	19.87	3118	31.34	
60-69	217	28.37	3858	38.78	
≥70	325	42.48	746	7.5	
**BMI** (kg/m2)					<.0001
<18.5	31	4.16	277	2.79	
18.5-24	293	39.33	4927	49.56	
24-27	223	29.93	2761	27.77	
≥27	198	26.58	1976	19.88	
Missing	20		8		
**Serum ALT** (IU/L)					<.0001
<15	65	8.53	2721	27.35	
15-45	495	64.96	6554	65.88	
≥45	202	26.51	674	6.77	
Missing	3		0		<.0001
**Serum AFP** (ng/mL)					
<5	260	34.35	8881	89.27	
≥5	497	65.65	1068	10.73	
Missing	8		0		<.0001
**Alcohol consumption**					
Never	406	54.35	9043	90.9	
Ever	341	45.65	905	9.1	
Missing	18		1		
**Cigarette smoking**					<.0001
Never	350	46.85	8312	83.55	
Ever	397	53.14	1637	16.45	
Missing	18		0		
**Fatty liver†**					<.0001
No	623	81.44	5607	56.40	
Yes	142	18.56	4335	43.60	
Missing	0		7		

† Fatty liver: Diagnosed by abdominal ultrasonography.

# Abbreviations: HCC, hepatocellular carcinoma. BMI, body mass index. ALT, alanine aminotransferase. AFP, alpha-fetoprotein.

Table S2. Frequency distribution of the 10 HCC-associated SNPs in the GWAS discovery sets.

		**HCC** (n=765)		**non-HCC control** (n=9949)		
**SNP**	**A1A2**	n	%	n	%	**P-value**
PNPLA3						
**rs738409**	CC	240	31.37	3714	37.33	<.0001
	CG	347	45.36	4813	48.38	
	GG	178	23.27	1422	14.29	
rs738408	CC	241	31.50	3728	37.47	<.0001
	TC	347	45.36	4805	48.30	
	TT	177	23.14	1416	14.23	
rs3747207	GG	246	32.16	3790	38.09	<.0001
	GA	348	45.49	4780	48.05	
	AA	171	22.35	1379	13.86	
rs4823173	GG	242	31.63	3682	37.01	<.0001
	GA	345	45.10	4822	48.47	
	AA	178	23.27	1445	14.52	
**rs2281135**	GG	232	30.33	3608	36.26	<.0001
	GA	352	46.01	4846	48.71	
	AA	181	23.66	1495	15.03	
rs2896019	TT	231	30.20	3604	36.22	<.0001
	TG	354	46.27	4845	48.70	
	GG	180	23.53	1500	15.08	
rs2294915	CC	230	30.07	3595	36.13	<.0001
	TC	353	46.14	4845	48.70	
	TT	182	23.79	1509	15.17	
SAMM50						
**rs2235776**	CC	233	30.46	3652	36.71	<.0001
	TC	360	47.06	4850	48.75	
	TT	172	22.48	1447	14.54	
rs2143571	GG	231	30.20	3616	36.35	<.0001
	GA	359	46.93	4861	48.86	
	AA	175	22.88	1472	14.80	
rs2073080	CC	230	30.07	3614	36.33	<.0001
	TC	361	47.19	4864	48.89	
	TT	174	22.75	1471	14.79	

Abbreviations: HCC, hepatocellular carcinoma. A1A2, genotype.

Table S3. HCC risk by genotypes of the 3 HCC-associated SNPs in the GWAS discovery stratified by the presence of fatty liver.

SNP	Genotype	Without Fatty Liver† (5607 controls, 623 cases)		With Fatty Liver† (4335 controls, 142 cases)	
		**Crude OR (95% CI)**	**Multivariate adjusted OR¶ (95% CI)**	**Crude OR (95% CI)**	**Multivariate adjusted OR¶ (95% CI)**
**rs738409**	CC	1.00 (reference)	1.00 (reference)	1.00 (reference)	1.00 (reference)
	CG	1.17 (0.97-1.41)	1.12 (0.83-1.50)	1.07 (0.72-1.62)	1.12 (0.67-1.86)
	GG	2.21 (1.74-2.79)	2.32 (1.58-3.43)	2.13 (1.37-3.32)	2.10 (1.18-3.74)
		P_{trend} <.0001	P_{trend} =0.0002	P_{trend} =0.0016	P_{trend} =0.0157
**rs2281135**	GG	1.00 (reference)	1.00 (reference)	1.00 (reference)	1.00 (reference)
	GA	1.17 (0.97-1.42)	1.16 (0.86-1.55)	1.12 (0.74-1.70)	1.25 (0.74-2.09)
	AA	2.11 (1.67-2.67)	2.04 (1.38-3.01)	2.14 (1.37-3.35)	2.31 (1.29-4.13)
		P_{trend} <.0001	P_{trend} =0.0012	P_{trend} =0.0013	P_{trend} =0.0058
**rs2235776**	CC	1.00 (reference)	1.00 (reference)	1.00 (reference)	1.00 (reference)
	TC	1.21 (1.00-1.46)	1.28 (0.95-1.72)	1.15 (0.77-1.72)	1.12 (0.68-1.86)
	TT	2.18 (1.72-2.76)	2.16 (1.46-3.20)	1.83 (1.16-2.88)	1.71 (0.95-3.10)
		P_{trend} <.0001	P_{trend} =0.0003	P_{trend} =0.0136	P_{trend} =0.0887

† Fatty liver: Diagnosed by abdominal ultrasonography.

¶ Adjusted for the first 20 principal components, age, sex, BMI, cigarette smoking, alcohol consumption, serum ALT levels, and serum AFP levels.

# Abbreviations: HCC, hepatocellular carcinoma. A1A2, genotype. OR, odds ratio. CI, confidence interval.

Table S4. Odds ratios of the association of the three SNPs with HCC among cirrhotic patients.

	GWAS discovery phase (152 HCC cases, 52 non-HCC controls)		Validation phase Hospital-based case-control study (104 HCC cases, 138 non-HCC controls)	
**SNP**	Crude OR (95%)	Multi-adjusted* OR (95%)	Crude OR (95%)	Multi-adjusted§ OR (95%)
**rs738409**				
CC	1.00 (reference)	1.00 (reference)	1.00 (reference)	1.00 (reference)
GC	4.33 (2.07-9.08)	6.02 (1.79-20.24)	1.46 (0.79-2.72)	1.45 (0.76-2.77)
GG	10.13 (3.81-26.9)	12.39 (2.80-54.87)	3.22 (1.63-6.47)	3.06 (1.52-6.31)
P trend	<.0001	0.0006	<.0001	0.0020
**rs2281135**				
GG	1.00 (reference)	1.00 (reference)	1.00 (reference)	1.00 (reference)
AG	4.27 (2.04-8.95)	5.51 (1.67-16.31)	1.51 (0.82-2.83)	1.44 (0.76-2.78)
AA	10.31 (3.88-27.39)	10.99 (2.56-47.27)	3.06 (1.53-6.27)	2.93 (1.42-6.15)
P trend	<.0001	0.0008	0.0020	0.0040
**rs2235776**				
CC	1.00 (reference)	1.00 (reference)	1.00 (reference)	1.00 (reference)
TC	3.80 (1.87-7.71)	6.40 (1.95-20.94)	1.58 (0.86-2.93)	1.49 (0.80-2.81)
TT	18.53 (5.21-65.91)	25.49 (4.34-149.54)	2.31 (1.14-4.75)	2.22 (1.06-4.71)
P trend	<.0001	0.0001	0.0200	0.0350

*Adjusted for sex, age, BMI, smoking, alcohol, ALT, and AFP.
§ Adjusted for sex, age, BMI, ALT, and diabetes.
# Abbreviations: HCC, hepatocellular carcinoma. OR, odds ratio. CI, confidence interval.

Table S5. Baseline characteristics of the study population in the validation sets.

Baseline characteristics	Community-based set (N=855)				Hospital-based set (N=5930)			
	**HCC** (n=171)		**non-HCC control** (n=684)		**HCC** (n=470)		**non-HCC control** (n=5460)	
	n	%	n	%	n	%	n	%
**Sex**								
Male	103	60.2	412	60.2	298	63.4	2533	46.4
Female	68	39.8	272	39.8	172	36.6	2927	53.6
**Age** (years)								
30-39	16	9.4	63	9.2	17	3.6	507	9.3
40-49	38	22.2	151	22.1	36	7.7	864	15.8
50-59	71	41.5	286	41.8	102	21.7	1239	22.7
60-69	46	26.9	184	26.9	140	29.8	1547	28.3
≥70	0		0		175	37.2	1303	37.2
**BMI** (kg/m^2)								
<18.5	10	5.8	18	2.6	24	5.1	224	4.1
18.5-24	59	34.5	315	46.1	175	37.2	2128	39.0
24-27	50	29.2	217	31.7	129	27.4	1481	27.1
≥27	52	30.4	134	19.6	142	30.2	1627	29.8
**Serum ALT** (IU/L)								
<15	98	57.3	463	67.7	67	14.3	1071	19.6
15-45	61	35.7	203	29.7	290	61.7	3275	60.0
≥45	12	7.0	18	2.6	113	24.0	1114	20.4
**Alcohol consumption**								
Never	137	80.1	615	90.0				
Ever	34	19.9	68	10.0				
Missing	0		1					
**Cigarette smoking**								
Never	102	59.6	470	68.7				
Ever	69	40.4	214	31.3				

Abbreviations: HCC, hepatocellular carcinoma. BMI, body mass index. ALT, alanine aminotransferase.

Table S6. HCC risk by genotypes of the 3 HCC-associated SNPs in the GWAS validation stratified by the BMI.

		**Hospital-based set** (470 cases, 5460 controls)			
		BMI < 24 $^{kg}/_{m}{}^{2}$ (199 cases, 2352 controls)		BMI ≥ 24 $^{kg}/_{m}{}^{2}$ (271 cases, 3108 controls)	
**SNP**	**Genotype**	**Crude OR (95% CI)**	**Multivariate adjusted OR¶ (95% CI)**	**Crude OR (95% CI)**	**Multivariate adjusted OR¶ (95% CI)**
**rs738409**	CC	1.00 (reference)	1.00 (reference)	1.00 (reference)	1.00 (reference)
	GC	1.23 (0.88, 1,73)	1.25 (0.89, 1.76)	0.95 (0.71, 1.28)	0.98 (0.73, 1.32)
	GG	1.82 (1.20, 2.73)	1.73 (1.13, 2.62)	1.89 (1.37, 2.63)	1.93 (1.38, 2.69)
		P_{trend} =0.006	P_{trend} =0.012	P_{trend} <0.001	P_{trend} <0.001
**rs2281135**	GG	1.00 (reference)	1.00 (reference)	1.00 (reference)	1.00 (reference)
	AG	1.21 (0.86, 1.69)	1.21 (0.86, 1.71)	0.94 (0.70, 1.27)	0.96 (0.71, 1.30)
	AA	1.62 (1.07, 2.43)	1.54 (1.01, 2.33)	1.82 (1.32, 2.53)	1.87 (0.34, 2.60)
		P_{trend} =0.024	P_{trend} =0.047	P_{trend} <0.001	P_{trend} <0.001
**rs2235776**	CC	1.00 (reference)	1.00 (reference)	1.00 (reference)	1.00 (reference)
	TC	1.27 (0.91, 1.78)	1.28 (0.92, 1.81)	1.05 (0.79, 1.42)	1.06 (0.79, 1.43)
	TT	1.46 (0.95, 2.21)	1.39 (0.89, 2.13)	1.70 (1.22, 2.38)	1.75 (1.24, 2.45)
		P_{trend} =0.065	P_{trend} =0.105	P_{trend} =0.004	P_{trend} =0.003

¶ Adjusted for sex, age, serum ALT levels, and diabetes.

# Abbreviations: HCC, hepatocellular carcinoma. BMI, body mass index. OR, odds ratio. CI, confidence interval.

Table S7. Baseline characteristics of the study population in the prospective cohort.

Baseline characteristics	Total (N=67909) n	%	HCC (n=32) n	%	non-HCC control (n=67877) n	%	P-value
**Sex**							<.0001
Male	22514	33.2	11	34.4	22503	33.2	
Female	45395	66.8	21	65.6	45374	66.9	
**Age** (years)							<.0001
30-39	17271	25.4	0	0	17271	25.4	
40-49	16419	24.2	3	9.4	16416	24.2	
50-59	18738	27.6	8	25.0	18730	27.6	
60-69	15183	22.4	20	62.5	15163	22.3	
≥70	298	0.4	1	3.1	297	0.4	
**BMI** (kg/m^2)							0.0021
<18.5	2429	3.6	0	0	2429	3.6	
18.5-24	33123	48.8	6	18.8	33117	48.8	
24-27	17988	26.5	15	46.9	17973	26.5	
≥27	14369	21.2	11	34.4	14358	21.2	
**Serum ALT** (IU/L)							<.0001
<15	21913	32.3	5	15.6	21908	32.3	
15-45	40649	59.9	18	56.3	40631	59.9	
≥45	5344	7.9	9	28.1	5335	7.9	
Missing	3		0		3		
**Alcohol consumption**							<.0001
Never	62170	91.6	22	68.8	62148	91.6	
Ever	5693	8.4	10	31.3	5683	8.4	
Missing	46		0		46		
**Cigarette smoking**							0.0018
Never	54978	81.0	19	59.4	54959	81.0	
Ever	12904	19.0	13	40.6	12891	19.0	
Missing	27		0		27		

Abbreviations: HCC, hepatocellular carcinoma. BMI, body mass index. ALT, alanine aminotransferase.

Table S8. Validation of the HLA signal from GWAS results in the prospective cohort.

	**Total** (2n=135818)		**Control** (2n=135754)		**HCC** (2n=64)		
HLA-DPB1	2n	%	2n	%	2n	%	**P-value**
DPB1*02:01	21486	15.8	21476	15.8	10	15.6	0.9659
DPB1*02:02	10074	7.4	10070	7.4	4	6.3	0.1887
DPB1*04:01	10987	8.1	10984	8.1	3	4.7	0.1285
DPB1*05:01	59547	43.8	59513	43.8	34	53.1	0.1344
DPB1*13:01	8644	6.4	8638	6.4	6	9.4	0.3237
Rare (freq<5% mix)	25080	18.5	25073	18.5	7	10.9	0.1205
