# Supplementary material for: Genome-wide association study identifies three PNPLA3/SAMM50 SNPs associated with HCC development in non-viral liver disease
Source: JHEP Rep. 2025 Nov 11;8(2):101673. doi: 10.1016/j.jhepr.2025.101673 (PMC12857342; doi:10.1016/j.jhepr.2025.101673)
Supplement: Multimedia component 2 [file mmc2.docx]

**JHEP Reports**

**CTAT methods**

Tables for a “Complete, Transparent, Accurate and Timely account” (CTAT) are now mandatory for all revised submissions. The aim is to enhance the reproducibility of methods.

- Only include the parts relevant to your study
- Refer to the CTAT in the main text as ‘Supplementary CTAT Table’
- Do not add subheadings
- Add as many rows as needed to include all information
- Only include one item per row

**If the CTAT form is not relevant to your study, please outline the reasons why:**

|  |
| --- |

- 1. **Antibodies**

| **Name** | **Citation** | **Supplier** | **Cat no.** | **Clone no.** |
| --- | --- | --- | --- | --- |
| Not applicable | | | | |

- 1. **Cell lines**

| **Name** | **Citation** | **Supplier** | **Cat no.** | **Passage no.** | **Authentication test method** |
| --- | --- | --- | --- | --- | --- |
| Not applicable | | | | | |

- 1. **Organisms**

| **Name** | **Citation** | **Supplier** | **Strain** | **Sex** | **Age** | **Overall n number** |
| --- | --- | --- | --- | --- | --- | --- |
| Not applicable | | | | | | |

- 1. **Sequence based reagents**

| **Name** | **Sequence** | **Supplier** |
| --- | --- | --- |
| Not applicable | | |

- 1. **Biological samples**

| **Description** | **Source** | **Identifier** |
| --- | --- | --- |
| Not applicable | | |

- 1. **Deposited data**

| **Name of repository** | **Identifier** | **Link** |
| --- | --- | --- |
| Taiwan Biobank (TWB) | TWBR11104-1 | https://www.twbiobank.org.tw/ |
| Health and Welfare Data Science Center Database, Ministry of Health and Welfare (NHIRD_MOHW) | H112006 | https://www.apre.mohw.gov.tw/ |

- 1. **Software**

| **Software name** | **Manufacturer** | **Version** |
| --- | --- | --- |
| SAS | SAS Institute Inc., Cary, NC | 9.4 |
| PLINK | Shaun Purcell & Broad Institute (open-source, GPL v3 license) | 1.9 |
| R | RStudio, Inc. | 4.3.2 |
| Haploview | Mark Daly's lab at the Broad Institute | 4.2 |

- 1. **Other (*e.g*. drugs, proteins, vectors etc.)**

| Axiom™ Genome-Wide TWB 2.0 Array (Thermo Fisher Scientific) |
| --- |

- 1. **Please provide the details of the corresponding methods author for the manuscript:**

| Mei-Hsuan Lee  Institute of Clinical Medicine, National Yang Ming Chiao Tung University  155 Li-Nong Street, Section 2, Beitou, Taipei 112, Taiwan  Tel: +886-2-2826-7248  Fax: +886-2-2820-5699  E-mail: meihlee@nycu.edu.tw |
| --- |

**2.0 Please confirm for randomised controlled trials all versions of the clinical protocol are included in the submission. These will be published online as supplementary information.**

| Not applicable |
| --- |
