# Supplementary material for: Genome-wide association study identifies three PNPLA3/SAMM50 SNPs associated with HCC development in non-viral liver disease
Source: JHEP Rep. 2025 Nov 11;8(2):101673. doi: 10.1016/j.jhepr.2025.101673 (PMC12857342; doi:10.1016/j.jhepr.2025.101673)
Supplement: Multimedia component 4 [file mmc4.pdf]

Research article

JHEP|Reports

Genome-wide association study identifies three PNPLA3/SAMM50 SNPs associated with HCC development in non-viral liver disease

Authors

Xia-Rong Liu, Tsai-Hsuan Yang, Tung-Hung Su, …, Chien-Jen Chen, Jia-Horng Kao, Mei-Hsuan Lee

Correspondence

meihlee@nycu.edu.tw (M.-H. Lee).

Graphical abstract

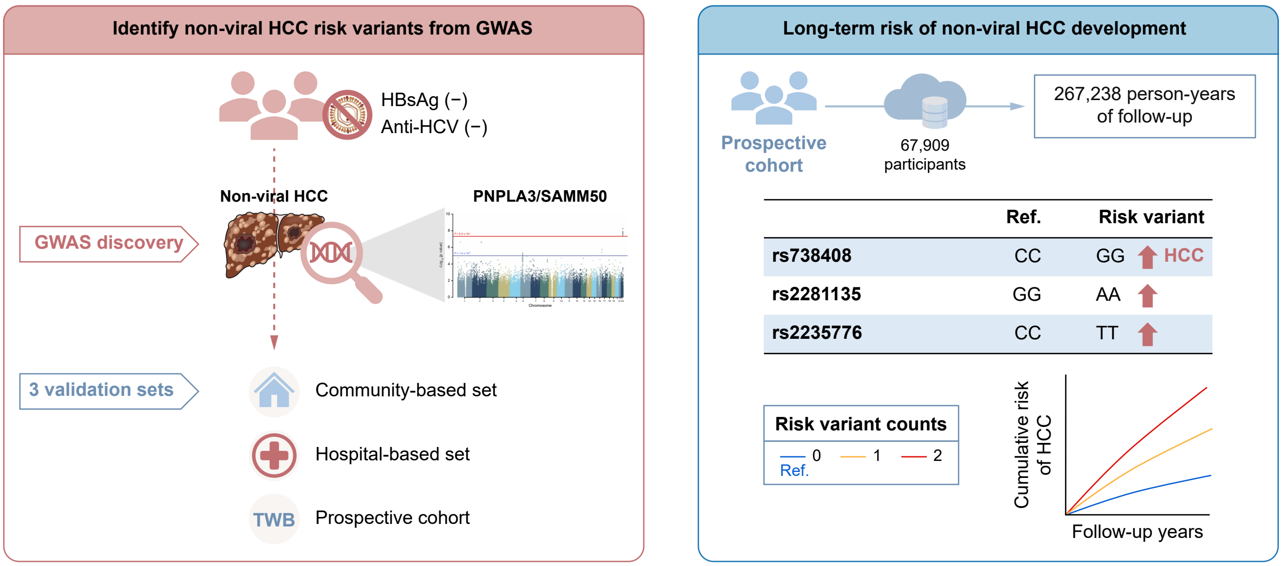

Highlights:

- Multi-stage GWAS identified HCC risk variants in HBsAg and anti-HCV seronegative individuals.
- *PNPLA3/SAMM50* variants were associated with HCC, independent of steatotic liver disease.
- Findings were validated in a hospital case-control and community cohort, showing robustness and relevance.
- *PNPLA3/SAMM50* were associated with long-term HCC risk and may guide non-viral risk stratification.

Impact and implications:

Variants in the *PNPLA3/SAMM50* locus were significantly associated with hepatocellular carcinoma (HCC) risk in individuals seronegative for HBsAg and anti-HCV. The stepwise increase in HCC risk with higher numbers of risk alleles suggests their potential utility as biomarkers to identify high-risk individuals without a history of chronic hepatitis B or C virus infection. These findings provide a foundation for genetic risk stratification and precision prevention strategies in populations where non-viral etiologies of HCC are becoming increasingly important.

https://doi.org/10.1016/j.jhepr.2025.101673

© 2025 The Authors. Published by Elsevier B.V. on behalf of European Association for the Study of the Liver (EASL). This is an open access article under the CC BY license (http://creativecommons.org/licenses/by/4.0/). JHEP Reports, 2026, 8, 1–10

JHEP|Reports

Genome-wide association study identifies three PNPLA3/SAMM50 SNPs associated with HCC development in non-viral liver disease

Xia-Rong Liu[1,†], **Tsai-Hsuan Yang**[1,†], Tung-Hung Su[2,3], Szu-Ching Yin[1], Yi-Ting Chen[1], Fen-Fang Chen[2], See-Tong Pang[4,5], Ming-Chih Hou[6], Yen-Chun Peng[7,8,9], Shun-Fa Yang[10,11], Peng-Ju Huang[12,13], Sing-Lian Lee[14], Ming Chen[15,16,17], Chih-Yang Huang[18,19,20,21,22], Ya-Hsuan Chang[23], Hsuan-Yu Chen[24], Hwai-I Yang[25], Ming-Lung Yu[13,26,27], Chien-Jen Chen[25], Jia-Horng Kao[2,3,28], Mei-Hsuan Lee[1,27,29,*]

JHEP Reports **2026**. vol. 8 | 1–10

Check for updates

Background & Aims: Few genome-wide association studies have examined genetic variants associated with hepatocellular carcinoma (HCC) risk in individuals seronegative for HBsAg and anti-HCV, and the long-term impact of these variants remains uncertain.

Methods: This multi-stage study analyzed adults >30 years old who were seronegative for HBsAg and anti-HCV. In the genome-wide association study discovery phase, 765 HCC cases and 9,949 controls were analyzed for 308,693 SNPs, with significant SNPs confirmed in community-based (171 HCC cases, 684 controls) and hospital-based (470 HCC cases, 5,460 controls) validation sets. A cohort of 67,909 participants, followed from 2012 to 2021, was used to evaluate the long-term HCC risk associated with these variants.

Results: Ten SNPs in *PNPLA3/SAMM50* were significantly associated with HCC risk (p <1.62 × 10^{-7}) and were in high linkage disequilibrium. Three SNPs (rs738409, rs2281135, rs2235776) were replicated and demonstrated strong associations with HCC, independent of steatosis. Over 267,238 person-years of follow-up, 32 new HCC cases occurred, with elevated risks observed in individuals homozygous for the risk genotypes: GG (rs738409), AA (rs2281135), and TT (rs2235776). The adjusted hazard ratios (95% CI) were 3.37 (1.32–8.63), 2.80 (1.06–7.37), and 2.64 (0.91–7.66), respectively. In allelic models, carriers of the risk allele had higher HCC risk, with adjusted hazard ratios (95% CI) of 1.88 (1.14–3.10) for rs738409, 1.68 (1.02–2.78) for rs2281135, and 1.61 (0.98–2.67) for rs2235776.

Conclusions: This study identified *PNPLA3/SAMM50* variants significantly associated with long-term HCC risk in individuals without viral hepatitis. These findings highlight their potential utility as biomarkers for HCC risk stratification and underscore the need for further research into underlying mechanisms.

© 2025 The Authors. Published by Elsevier B.V. on behalf of European Association for the Study of the Liver (EASL). This is an open access article under the CC BY license (http://creativecommons.org/licenses/by/4.0/).

Introduction

Hepatocellular carcinoma (HCC) is the sixth most common malignancy worldwide and a leading cause of cancer-related deaths, with over 50% of global HCC-related deaths occurring in the Asia-Pacific region.[1] Although chronic hepatitis B and C virus (HBV and HCV) infections are major contributors to HCC cases, its development is multifactorial.[2] With the widespread implementation of hepatitis B vaccination programs and the availability of highly effective antivirals for hepatitis C, the etiology of HCC is evolving. Increasingly, HCC cases are arising in individuals without chronic hepatitis B or C virus infections, presenting a growing public health challenge in the coming years.

Familial clustering of HCC[3,4] has suggested a substantial heritable component to its development, indicating that genetic predisposition is a significant determinant. HCC risk may be influenced by multiple genetic variants,[5] making genome-wide association studies (GWAS) a powerful tool to identify common genetic variants linked to HCC susceptibility. However, most prior GWAS on HCC have primarily focused on populations with chronic HBV[6,7] or HCV[8–11] infections. While some GWAS have investigated HCC unrelated to hepatitis B or C, these studies have often concentrated on alcohol-related HCC[12,13] or lacked clear definitions of HBV and HCV infection status,[14–16] leading to potential population heterogeneity. Consequently, GWAS specifically examining

* Corresponding author. Address: Institute of Clinical Medicine, National Yang Ming Chiao Tung University, 155 Li-Nong Street, Section 2, Beitou, Taipei 112, Taiwan; Tel.: +886-2-2826-7248, fax: +886-2-2820-5699.
E-mail address: meihlee@nycu.edu.tw (M.-H. Lee).
† These authors contributed equally to this work
https://doi.org/10.1016/j.jhepr.2025.101673

HCC risk among individuals without chronic HBV or HCV infections are limited, and the long-term impact of identified variants on HCC risk remained unclear.

To address this gap, we conducted a large-scale GWAS to identify genetic variants associated with HCC in individuals seronegative for hepatitis B surface antigen (HBsAg) and antibodies against hepatitis C virus (anti-HCV). We validated the identified single nucleotide polymorphisms (SNPs) in independent community- and hospital-based case-control studies. Finally, we conducted a follow-up study to assess the long-term impact of these SNPs on HCC.

Patients and methods

Study design and flow

This study included only participants aged 30 years or older who were seronegative for HBsAg and anti-HCV. Both case-control and prospective cohort designs were used, as illustrated in Fig. 1.

GWAS discovery phase

In the first stage, a GWAS was conducted to compare 765 HCC cases with 9,949 non-HCC controls. The HCC cases were sourced from the Taiwan Liver Cancer Network (TLCN), and the controls were drawn from the Taiwan Biobank.

Validation phase

In the second stage, SNPs identified as significantly associated with HCC risk in the GWAS phase were validated in two independent case-control studies: a community-based set (171 HCC cases and 684 controls) and a hospital-based set from the TPMI (Taiwan Precision Medicine Initiative) (470 HCC cases and 5,460 controls). Finally, we assessed the long-term risk of HCC associated with these SNPs in a prospective cohort of 67,909 Taiwan Biobank participants, all distinct from the controls used in the GWAS phase. This cohort was followed over time to determine HCC risk. Neither patients nor

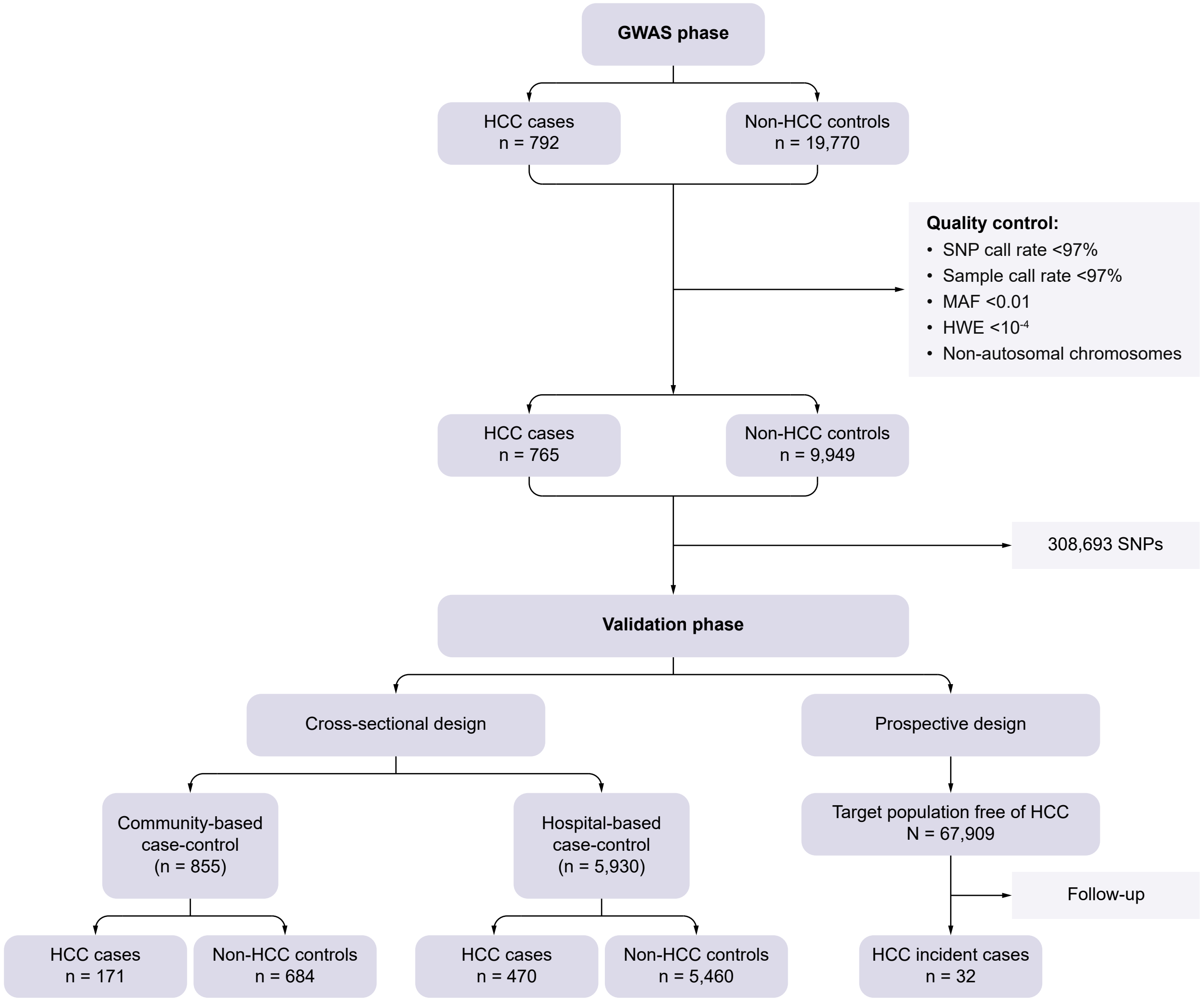

Fig. 1. Study design and participant flow diagram. HCC, hepatocellular carcinoma; HWE, Hardy-Weinberg equilibrium; MAF, minor allele frequency; SNP, single nucleotide polymorphism.

the public were involved in the design, conduct, reporting, or dissemination of this research. The study protocol was approved by the Institutional Review Board of National Yang Ming Chiao Tung University in Taipei, Taiwan.

TLCN

The TLCN is a national biobank comprising biological specimens from patients with HCC treated at major medical centers across Taiwan. Informed consent was obtained for sample collection, epidemiological data, abdominal ultrasound information, and virological analysis. Sample collection and handling were standardized, and all samples were stored at −80 °C until further analysis.[8,17]

Taiwan Biobank

The Taiwan Biobank is a government-approved repository that enrolls participants from the general Taiwanese population. Collected data include personal and family history questionnaires, blood and urine biochemical tests, and biological samples such as DNA, plasma, and urine. To date, 189,108 participants have been enrolled, most of whom have undergone whole-genome SNP genotyping. Participants from the Taiwan Biobank were included as controls in the GWAS phase (n = 9,949) and as the prospective cohort in the replication phase (n = 67,909), with no overlap between the two groups.

To ascertain HCC cases and participants' status, we linked computerized data across three nationwide registries maintained by Taiwan's Ministry of Health and Welfare, covering the period from January 1, 2012, to December 31, 2021. HCC cases were identified using ICD codes 155 (ICD-9) and C22 (ICD-10) from the National Cancer Registry and confirmed through histopathology, imaging, or elevated serum alpha-fetoprotein (AFP) levels.

Community-based case-control validation set

The community-based cohort, previously described, comprised 18,541 individuals seronegative for HBsAg and anti-HCV from a baseline pool of 23,820 participants across seven Taiwanese townships.[18,19] Blood samples were collected, processed, and stored at −70 °C for analysis. During follow-up, 171 cases of HCC were identified, and control groups matched by age and sex in a 1:4 ratio were randomly selected. The SNPs identified in the GWAS phase were genotyped using the TaqMan genotyping assay (Applied Biosystems).

Hospital-based case-control validation set

The TPMI is a large-scale genetic project involving volunteers from 16 medical centers across Taiwan.[20] For this study, 470 HCC cases and 5,460 controls with sufficient clinical and genotypic data were included, with information on age, sex, BMI, and serum alanine aminotransferase (ALT) levels. Diabetes was identified using ICD codes 250 (ICD-9) and E11 (ICD-10). SNP genotyping was performed using an adapted SNP array from the Taiwan Biobank 2.0 Array Plate.

SNP genotyping and quality control

Genomic DNA was extracted from peripheral blood leukocytes using standard protocols. Genotyping for the GWAS discovery phase used the Axiom™ Genome-Wide TWB 2.0 Array (Thermo Fisher Scientific). Quality control (QC) steps removed SNPs with call rates <97%, minor allele frequencies <1%, or those violating Hardy-Weinberg equilibrium ($p < 10^{-4}$). Samples with call rates <97%, discrepancies between self-reported and genetically determined sex, and heterozygosity rates more than three standard deviations from the mean were excluded. Identity-by-descent analysis removed cryptic relatedness between subject pairs with identity-by-descent scores >0.1875. After QC, 308,693 SNPs remained for analysis.

Statistical methods

Principal component analysis (PCA) was conducted to assess population structure, with the first two principal components plotted. Quantile-quantile (Q-Q) plots and the genomic inflation factor (λ) were used to assess the degree of population stratification. SNPs surpassing the Bonferroni-corrected significance threshold of 1.62×10^{-7} were considered genome-wide significant. A Manhattan plot depicted the $-\log_{10}$ (p values) for all SNPs passing QC. Further analysis and visualization were conducted using R and PLINK, with LocusZoom and HAPLOVIEW employed for regional association and linkage disequilibrium analysis. We selected three highly linked tag SNPs for validation analyses. Logistic regression models, adjusting for age, sex, BMI, serum ALT and AFP levels, smoking, and alcohol use, estimated odds ratios (ORs), and 95% CIs. Adjustments were made for potential population stratification using PCA. Ultrasound data provided steatosis status for all study participants (765 HCC and 9,949 controls) in the GWAS phase, allowing for stratified SNP-HCC association analysis by steatosis status. In the validation phase, BMI was used as a steatosis proxy in the hospital cohort.

In the prospective analysis, Cox proportional hazard models were used to evaluate the association between SNP genotypes and HCC risk over time. Kaplan-Meier survival curves and log-rank tests assessed cumulative HCC incidence across genotype groups. Hazard ratios (HRs) with 95% CIs quantified the associations between SNPs and HCC risk. Additionally, we integrated the three most significant SNPs identified and calculated the total number of risk alleles for each participant to estimate their cumulative risk for HCC. The Cochran-Armitage trend test was used to examine the dose-response relationship between the number of risk alleles and HCC risk across different genotypes. The proportional hazards assumption of the Cox models was tested and confirmed not to be violated. All statistical analyses were conducted using SAS version 9.4 (SAS Institute Inc., Cary, NC).

Results

GWAS of HCC in individuals seronegative for HBsAg and anti-HCV

In the GWAS discovery phase, HCC cases were older, more frequently male, and had higher BMI, serum ALT, and serum AFP levels than controls (<0.05). HCC cases also had a higher prevalence of smoking and alcohol consumption compared to controls (Table S1). Following quality control, 308,693 SNPs were retained for analysis across 765 HCC cases and 9,949 non-HCC controls. The Q-Q plot comparing observed *vs.* expected p values showed no early deviation, and the genomic

inflation factor (λ = 1.046) suggested minimal inflation, indicating limited population stratification. PCA of the first two components further confirmed tight clustering of cases and controls, mitigating concerns about confounding from population structure. PCA of the first two components revealed that cases and controls were tightly clustered, further confirming that population structure was unlikely to confound the findings.

The Manhattan plot, using an additive genetic model, identified a cluster of ten genome-wide significant SNPs on chromosome 22, with p values below the Bonferroni-corrected significance threshold of 1.62×10^{-7} (Fig. 2). Importantly, all ten SNPs identified on chromosome 22 remained significant under the conventional genome-wide threshold of $p < 5 \times 10^{-8}$[41,42] (Fig. 2), with p values ranging from 2.36×10^{-8} to 6.27×10^{-9}. This confirms that the associations are robust even under the more stringent criterion. To evaluate the association between genotype frequencies and HCC risk, individuals with complete genotype data for all ten genome-wide significant SNPs on chromosome 22 were included in the final analysis, comprising 765 HCC cases and 9,949 controls. Genotype frequencies were compared between cases and controls (Table S2).

HCC risk associated with *PNPLA3/SAMM50* genotypes

Among the ten significant SNPs identified on chromosome 22, three – rs738409, rs2281135, and rs2253776 in the *PNPLA3/SAMM50* gene – were selected for further analysis based on linkage disequilibrium patterns observed in HaploView (Fig. 2). The linkage disequilibrium plot demonstrated that these SNPs are in strong linkage disequilibrium (r^2 >0.8) with neighboring SNPs, suggesting that they belong to a risk locus spanning *PNPLA3* and *SAMM50*.

All three selected SNPs exhibited similar trends in their association with HCC risk. In the GWAS discovery phase, each SNP showed a significant association with increased HCC risk across various genotypes in both crude and adjusted models (Table 1). For rs738409, the heterozygous (CG) and homozygous (GG) genotypes were linked to increased HCC risk relative to the reference (CC) genotype, with adjusted ORs of 1.09 (95% CI 0.86-1.39) and 1.89 (95% CI 1.41-2.55), respectively (p_{trend} <0.0001). Similarly, for rs2281135, the GA and AA genotypes demonstrated a dose-dependent increase in risk compared to the GG genotype, with adjusted ORs (95% CI) of 1.14 (0.90-1.45) and 1.81 (1.34-2.43) (p_{trend} = 0.0003). Likewise, for rs2253776 in *SAMM50*, the TC and TT genotypes indicated high risk compared to the AA genotype, with adjusted ORs (95% CI) of 1.16 (0.92-1.48) and 1.73 (1.28-2.33) (p_{trend} = 0.0008). Stratification analyses by ultrasound-determined steatosis status, given previous associations of these SNPs with steatotic liver, revealed consistent SNP-HCC associations regardless of steatosis status (Table S3).

To assess whether the identified SNPs were associated with HCC independent of cirrhosis, we repeated the analyses restricted to participants with cirrhosis (Table S4). The associations remained significant (p <0.0001), suggesting that these variants represent HCC susceptibility loci rather than cirrhosis-related signals.

Cross-sectional validations of HCC-associated SNPs

Baseline characteristics of study participants in the community-based and hospital-based case-control validation sets are summarized in Table S4. In both sets, HCC cases had higher serum ALT levels and a greater history of smoking and alcohol use than controls (Table S4). We examined the magnitude of associations for the three target SNPs. The three SNPs in the hospital-based validation set showed significant associations with HCC, with adjusted ORs ranging from 1.60 to 1.85 for homozygous (Table 1). Stratified analyses, using BMI as a proxy for steatosis, revealed consistent associations between these SNPs and HCC in individuals with both BMI <24 kg/m^2 and BMI ≥24 kg/m^2 (Table S5). The associations remained significant across BMI strata, suggesting that the SNPs are associated with long-term HCC risk, independent of BMI. Although associations in the community-based validation set did not reach statistical significance, genotype patterns were consistent with those observed in the hospital-based set.

Long-term risk of HCC: Cumulative HCC risk by genotypes

To assess the long-term risk of HCC associated with three SNPs, we conducted a prospective validation study using data from the Taiwan Biobank. The baseline characteristics of the study population are displayed in Table S7. As illustrated in Fig. 3, the cumulative risks of HCC were stratified by the genotypes of rs738409, rs2281135, and rs2235776. Significant differences in the genotypes of these individual SNPs were observed in relation to HCC risk (p <0.001). In the GWAS discovery phase, we also observed a borderline cluster of SNPs in the *HLA* region. To further investigate this signal, we performed *HLA* imputation and examined the associations between imputed *HLA* genotypes and HCC risk; however, no significant associations were identified in the prospective validation analysis (Table S8).

HCC risk stratified by genotypes after follow-up

After 267,238 person-years of follow-up, 32 newly developed HCC were identified among the 67,909 study participants. As shown in Table 2, participants with homozygous genotype GG for rs738409, AA for rs2281135, and TT for rs2235776 had significantly increased risks of HCC, with adjusted HRs of 3.37 (95% CI 1.32–8.63), 2.80 (95% CI 1.06–7.37), and 2.64 (95% CI 0.91–7.66), respectively, compared to the reference genotypes. As shown in Table 3, allelic models further corroborated these findings, indicating increased HCC risk for individuals carrying risk alleles. The adjusted HRs (95% CI) were 1.88 (1.14–3.10) for the G allele of rs738409 compared to C, 1.68 (1.02–2.78) for the A allele of rs2281135 compared to G, and 1.61 (0.98–2.67) for the T allele of rs2235776 compared to C. Among the three SNPs tested, the rs738409 risk allele exhibited the strongest associations with HCC risk.

Discussion

We employed a multi-stage approach – starting with a GWAS for discovery, followed by validation in independent case-control sets and a prospective cohort – to enhance the reliability and reproducibility of our findings. This layered design helps minimize false positives and enhances the generalizability of the results. We identified a cluster of ten SNPs on chromosome 22, which are in high linkage disequilibrium, reinforcing the association between this region and HCC risk. Additionally, variants in *PNPLA3/SAMM50* were linked to long-

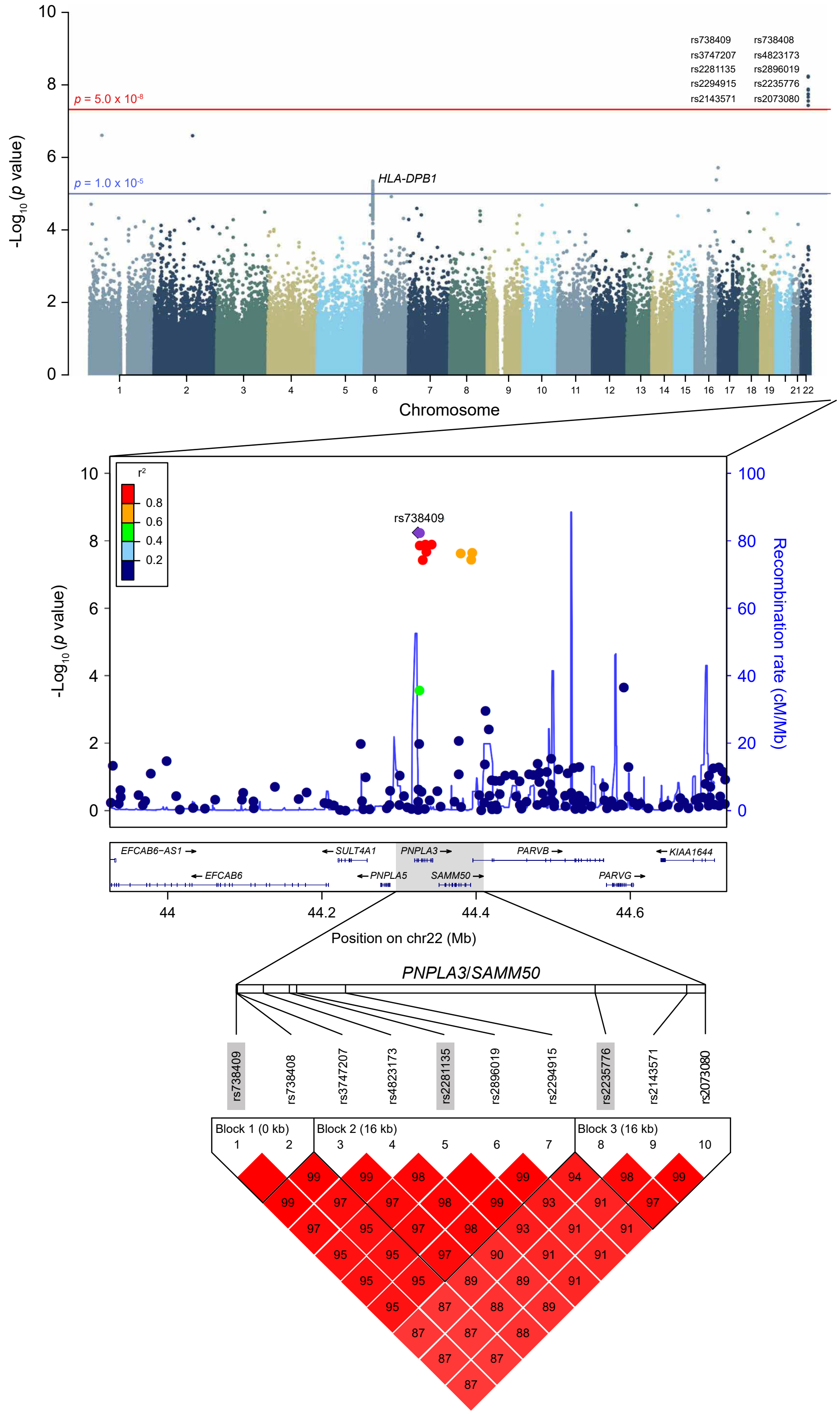

Fig. 2. Manhattan plot of HCC-associated SNPs in individuals seronegative for HBsAg and anti-HCV. *p* values were estimated using logistic regression models. The red line represents the conventional genome-wide significance threshold (p = 5×10^{-8}). HCC, hepatocellular carcinoma; SNP, single nucleotide polymorphism.

Table 1. Risk of HCC associated with SNPs in GWAS discovery and validation sets.

SNP	GWAS discovery phase: Study participants (765 HCC cases, 9,949 controls) Crude OR (95% CI)	Multivariate adjusted OR (95% CI)†	GWAS validation phase: Community-based case-control study (171 HCC cases, 684 controls) Crude OR (95% CI)	Multivariate adjusted OR (95% CI)§	Hospital-based case-control study (470 HCC cases, 5,460 controls) Crude OR (95% CI)	Multivariate adjusted OR (95% CI)¶
rs738409						
CC	1.00 (reference)	1.00 (reference)	1.00 (reference)	1.00 (reference)	1.00 (reference)	1.00 (reference)
CG	1.12 (0.94-1.32)	1.09 (0.86-1.39)	1.38 (0.94-2.02)	1.34 (0.90-1.99)	1.07 (0.85-1.33)	1.08 (0.87-1.36)
GG	1.94 (1.58-2.37)	1.89 (1.41-2.55)	1.46 (0.89-2.41)	1.38 (0.82-2.33)	1.88 (1.46-2.42)	1.85 (1.43-2.40)
	p_{trend} <0.0001	p_{trend} <0.0001	p_{trend} = 0.0888	p_{trend} = 0.154	p_{trend} <0.0001	p_{trend} <0.0001
rs2281135						
GG	1.00 (reference)	1.00 (reference)	1.00 (reference)	1.00 (reference)	1.00 (reference)	1.00 (reference)
GA	1.13 (0.95-1.34)	1.14 (0.90-1.45)	1.39 (0.95-2.04)	1.34 (0.90-1.99)	1.05 (0.84-1.32)	1.06 (0.84-1.33)
AA	1.88 (1.54-2.31)	1.81 (1.34-2.43)	1.33 (0.80-2.20)	1.26(0.75-2.12)	1.76 (1.36-2.26)	1.73 (1.34-2.25)
	p_{trend} <0.0001	p_{trend} = 0.0003	p_{trend} = 0.1672	p_{trend} = 0.266	p_{trend} <0.0001	p_{trend} <0.0001
rs2235776						
CC	1.00 (reference)	1.00 (reference)	1.00 (reference)	1.00 (reference)	1.00 (reference)	1.00 (reference)
TC	1.16 (0.98-1.38)	1.16 (0.92-1.48)	1.62 (1.10-2.39)	1.54 (1.03-2.30)	1.14 (0.92-1.43)	1.15 (0.92-1.44)
TT	1.86 (1.51-2.29)	1.73 (1.28-2.33)	1.60 (0.95-2.70)	1.43 (0.83-2.47)	1.61 (1.24-2.09)	1.60 (1.23-2.09)
	p_{trend} <0.0001	p_{trend} = 0.0008	p_{trend} = 0.0332	p_{trend} = 0.099	p_{trend} <0.0001	p_{trend} <0.0001

p_{trend} was calculate using the Cochran–Armitage test for trend. AFP, alpha-fetoprotein; ALT, alanine aminotransferase; HCC, hepatocellular carcinoma; OR, odds ratio.
†Adjusted for the first 20 principal components, age, sex, BMI, cigarette smoking, alcohol consumption, serum ALT levels, and serum AFP levels.
§Adjusted for age, sex, BMI, cigarette smoking, alcohol consumption, and serum ALT levels.
¶Adjusted for age, sex, BMI, serum ALT levels, and diabetes.

term HCC risk in individuals who were seronegative for both HBsAg and anti-HCV.

This study stratified participants based on the presence of steatotic liver disease, which had a prevalence of nearly 35% in the population.[21] Variants in *PNPLA3/SAMM50* were associated with steatotic liver susceptibility, suggesting that steatotic liver disease is an intermediate pathway in HCC development.[22] Mediation analyses would help determine whether these variants independently contribute to HCC risk or if steatotic liver mediates this relationship.[23] However, in both the GWAS discovery set (with ultrasound-based fatty liver data) and the hospital validation set (using obesity as a surrogate), we found that *PNPLA3/SAMM50* variants were significantly associated with HCC in participants with and without steatotic liver. These results indicate that the observed associations are not fully explained by fatty liver status. Notably, recent studies indicate that the *PNPLA3* variant rs738409 influences fibrosis independently of steatotic liver status,[23,24] suggesting that pathways beyond lipid accumulation may be involved in fibrogenesis and HCC risk.[23]

Our findings reveal that *PNPLA3/SAMM50* variants are associated with HCC risk across both steatotic and non-steatotic liver subgroups, underscoring potential pleiotropic effects[25] where these genes may contribute to HCC through diverse biological pathways beyond steatotic liver. By including participants from community-based cohorts, we were able to examine associations in individuals without steatotic liver disease, who might not otherwise be identified in clinical settings. These results underscore the importance of investigating these variants in broader populations to deepen our understanding of HCC risk factors. This approach may lay the groundwork for future multi-factorial risk prediction models in populations seronegative for HBsAg and anti-HCV.

As a non-synonymous SNP, rs738409 in the *PNPLA3* gene results in an isoleucine-to-methionine substitution (I148M).[26] This variant has been previously identified as a major susceptibility factor for metabolic dysfunction-associated steatotic liver disease (MASLD) in prior GWAS.[27–30] The mutant protein exhibits reduced lipolytic activity, leading to increased triglyceride accumulation in liver cells. *PNPLA3* encodes patatin-like phospholipase domain-containing protein 3, primarily expressed in adipose tissue and the liver, where it regulates lipid droplet turnover.[31] It is considered the largest contributor to genetic variability of hepatic fat accumulation in the general population.[27] Notably, a GWAS on patients with histologically confirmed MASLD identified *PNPLA3* as a marker for fibrosis severity,[29] suggesting a possible role in HCC development. Furthermore, the variant has been associated with cirrhosis risk,[32] highlighting its potential involvement in liver cancer progression. Beyond fibrosis, emerging evidence suggests more direct oncogenic roles.[39] It demonstrated that lipid stimulation of hepatocytes carrying the 148M variant induces pro-inflammatory and pro-angiogenic chemokines, including IL-8 and CXCL1, which promote migration and angiogenesis, thereby linking *PNPLA3* directly to tumor-promoting microenvironments. Consistent with this, our analysis showed that *PNPLA3* variants remained associated with HCC risk even among patients with cirrhosis, implying that their effect on HCC is not fully mediated by cirrhosis and may reflect pleiotropic mechanisms beyond fibrosis progression.

The precise relationship between *PNPLA3* variants and HCC risk remained incompletely understood. A meta-analysis of European patients with cirrhosis identified a significant association between the rs738409 variant and HCC, particularly in patients with alcohol-related liver disease.[33] Similarly, one case-control study reported that each copy of the rs738409 G allele was associated with an additive increase in HCC risk, with GG homozygotes exhibiting a fivefold higher risk.[22] Longitudinal studies specifically assessing the variant's contribution to HCC remained limited,[34–36] likely due to the relatively low HCC risk in patients with MASLD. Most studies aggregate multiple advanced liver-related outcomes, such as liver-specific death, transplantation, or decompensation, rather than focusing solely on incident HCC.[34–36] A recent prospective study found that *PNPLA3* variants were associated with an increased risk of major adverse liver outcomes in patients with

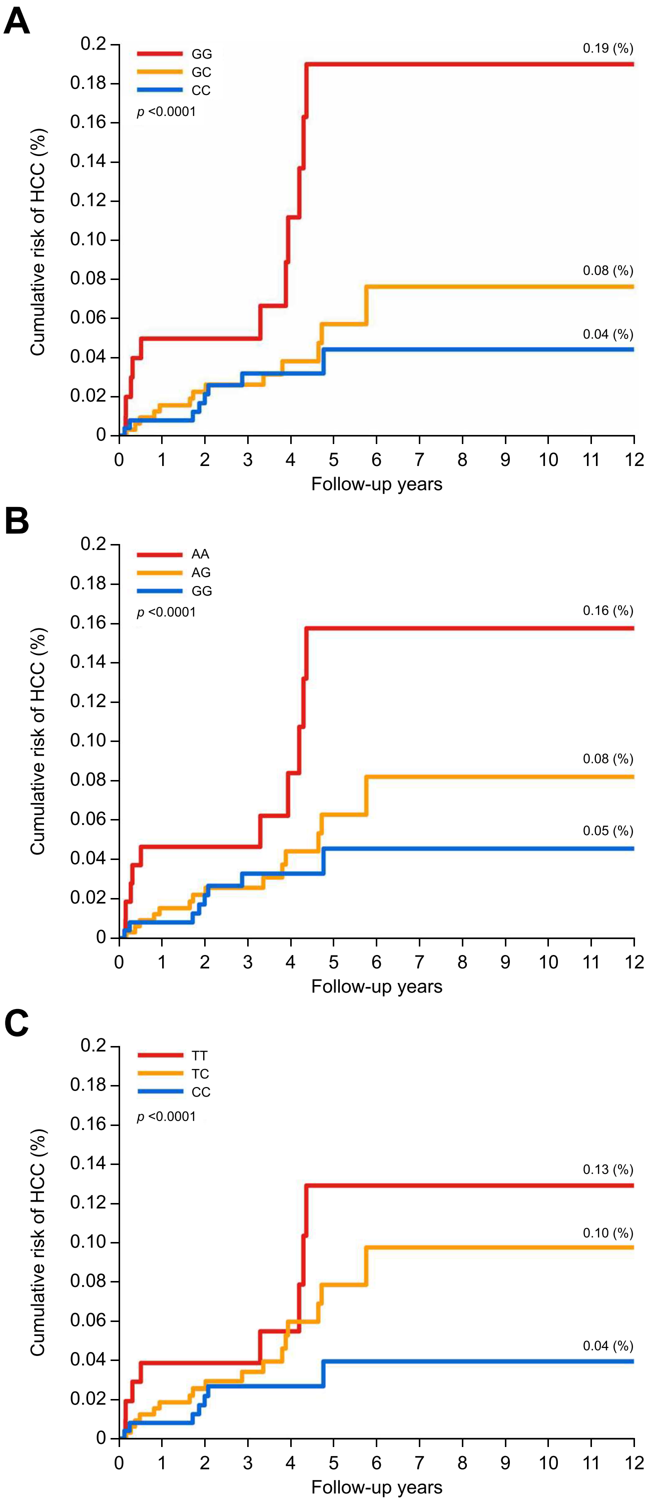

Fig. 3. Cumulative HCC risk stratified by genotypes in a prospective cohort (N = 67,909). (A) rs738409. (B) rs2281135. (C) rs2235776. The overall *p* value was calculated using the log-rank test. HCC, hepatocellular carcinoma.

steatosis, especially those with advanced fibrosis.[34] While another prospective study found no definitive impact on HCC due to the small number of cases, it highlighted that women aged 50 or older with MASLD carrying the rs738409 GG genotype were at high risk of severe liver outcomes.[36] These findings are consistent with our results, where rs738409 and rs2281135 variants in *PNPLA3* were associated with HCC in a dose-dependent manner based on the number of risk alleles carried. These findings suggest that the variant may serve as a genetic marker for identifying high-risk individuals, particularly those with advanced steatotic liver disease. Notably, a study of patients with alcohol-related cirrhosis reported that over 90% of individuals who carried *SAMM50* variants also carried the *PNPLA3* I148M allele, indicating that the *PNPLA3* I148M locus is a stronger predictor of alcohol-related HCC risk than genetic variation in *SAMM50*.[40] Because these variants are in strong linkage disequilibrium, we examined their associations through both allelic and genotypic models, which consistently demonstrated that increasing numbers of risk alleles corresponded to a stepwise increase in HCC risk. Taken together, these findings underscore the robustness of the *PNPLA3/SAMM50* locus as a susceptibility signal for HCC.

We also identified an association between the rs2235776 variant in the *SAMM50* gene and HCC risk. This SNP is in strong linkage disequilibrium with *PNPLA3/SAMM50*, which are often described as part of a single genomic block influencing MASLD susceptibility.[28] Beyond *PNPLA3*, *SAMM50* variants have been linked to HCC risk in recent GWAS.[14,16] *SAMM50* encodes Sam50, a protein involved in the mitochondrial outer membrane, responsible for inserting β-barrel proteins. Sam50 has been implicated in mitochondrial dysfunction, and its loss in hepatocytes induces mitochondrial membrane remodeling, leading to mitochondrial DNA release, liver inflammation, and subsequent liver injury.[37]

In comparison to previous GWAS on genetic variants and HCC risk,[14,16] our study uniquely focuses on individuals seronegative for HBsAg and anti-HCV, isolating risk factors not directly attributable to chronic hepatitis B or C infections in an Asian population undergoing a shift from viral to other etiologies. Previous studies,[14,16] such as those conducted in the US, included both hepatitis-related and unrelated HCC cases, potentially diluting associations specific to HCC risk outside of chronic hepatitis infections. Unlike prior studies, our multistage GWAS with prospective follow-up in a general population cohort allows us to assess cumulative HCC risk over time. Adjustments for BMI and lifestyle factors enhance our findings' relevance to populations facing rising metabolic HCC risks, underscoring the need for region-specific longitudinal studies to clarify evolving HCC risk profiles.

To our knowledge, this is the largest GWAS focused on individuals seronegative for both HBsAg and anti-HCV. We rigorously determined the virological status of all participants through comprehensive virological testing, ensuring a well-defined population. The study populations were homogeneous, consisting of Taiwanese individuals, which minimized confounding due to genetic diversity. Given that SNP-phenotype associations are often modest, our large sample size ensured adequate statistical power to detect significant associations between SNPs and HCC risk. By leveraging large biobanks and national registries, we obtained robust estimates of genetic associations. Linking to Taiwan's national cancer and death registries allowed us to comprehensively and accurately identify HCC cases. With a sufficiently long follow-up period, we were able to assess the impact of the identified

Table 2. Long-term HCC risk by genotypes of validated SNPs in a prospective cohort (N = 67,909).

SNP	Crude HR (95% CI)	Age-sex adjusted HR (95% CI)	Multivariate adjusted HR (95% CI)¶
rs738409			
CC	1.00 (reference)	1.00 (reference)	1.00 (reference)
GC	1.29 (0.54-3.11)	1.30 (0.54-3.13)	1.39 (0.57-3.38)
GG	3.53 (1.42-8.77)	3.70 (1.49-9.19)	3.37 (1.32-8.63)
	p_{trend} = 0.0087	p_{trend} = 0.0067	p_{trend} = 0.0144
rs2281135			
GG	1.00 (reference)	1.00 (reference)	1.00 (reference)
AG	1.33 (0.56-3.16)	1.33 (0.56-3.18)	1.42 (0.59-3.44)
AA	2.91 (1.15-7.37)	3.04 (1.20-7.70)	2.80 (1.06-7.37)
	p_{trend} = 0.0291	p_{trend} = 0.0236	p_{trend} = 0.0423
rs2235776			
CC	1.00 (reference)	1.00 (reference)	1.00 (reference)
TC	1.87 (0.78-4.51)	1.89 (0.79-4.56)	1.99 (0.82-4.87)
TT	2.75 (1.00-7.57)	2.91 (1.06-8.02)	2.64 (0.91-7.66)
	p_{trend} = 0.0461	p_{trend} = 0.0351	p_{trend} = 0.0600

p_{trend} was calculated using the Cochran-Armitage test for trend. AFP, alpha-fetoprotein; ALT, alanine aminotransferase; AST, aspartate aminotransferase; HbA1c, glycated hemoglobin; HCC, hepatocellular carcinoma; HR, hazard ratio.
¶Adjusted for age, sex, BMI, cigarette smoking, alcohol consumption, serum ALT levels, serum AST levels, serum AFP levels, and HbA1c.

Table 3. Allelic model for SNPs associated with HCC in a prospective cohort (N = 67,909).

SNP	AF in controls %	AF in cases %	Crude HR (95% CI)	*p* value	Age-sex adjusted HR (95% CI)	*p* value	Multivariate adjusted HR¶ (95% CI)	*p* value
rs738409								
C	61.4	45.3	1.00		1.00		1.00	
G	38.6	54.7	1.93 (1.18-3.16)	0.0089	1.97 (1.20-3.22)	0.0071	1.88 (1.14-3.10)	0.0139
rs2281135								
G	60.3	46.9	1.00		1.00		1.00	
A	39.7	53.6	1.73 (1.06-2.82)	0.0294	1.76 (1.08-2.87)	0.0244	1.68 (1.02-2.78)	0.0422
rs2235776								
C	60.7	48.4	1.00		1.00		1.00	
T	39.3	51.6	1.65 (1.01-2.69)	0.0459	1.69 (1.04-2.76)	0.0361	1.61 (0.98-2.67)	0.0616

HRs and *p* values were determined using Cox regression models. AF, allele frequency; ALT, alanine aminotransferase; AST, aspartate aminotransferase; HbA1c, glycated hemoglobin; HCC, hepatocellular carcinoma; HR, hazard ratio.
¶Adjusted for age, sex, BMI, cigarette smoking, alcohol consumption, serum ALT levels, serum AST levels, serum AFP levels, and HbA1c.

SNPs on HCC occurrence, which has rarely been explored in previous studies. Our community-based cohort allowed for the assessment of SNP-HCC associations beyond individuals with advanced liver disease,[35] thereby enhancing the clinical relevance of our findings for screening relatively healthy individuals for early diagnosis biomarkers.

However, there are some limitations to consider. HBV is endemic in Taiwan, with most chronic carriers acquiring the virus via vertical transmission. Many individuals in this population are positive for anti-HB core antibodies,[38] which may influence the findings. In our GWAS, we also observed a borderline cluster of SNPs in the *HLA* region, suggesting a potential residual viral or immune effect. This may reflect occult HBV infection, although its prevalence in the general population is relatively low. To more definitively evaluate the role of *HLA* variants in HCC unrelated to chronic HBV or HCV infection, larger studies with greater statistical power will be needed. While our results are relevant to populations in Asia where HBV is prevalent, generalizability to regions with different viral epidemiology needs further evaluation. Additionally, we lacked image-based data to diagnose steatotic liver disease in this cohort, which limited our ability to stratify analyses by steatotic liver status.

In conclusion, our study identified *PNPLA3/SAMM50* variants associated with increased HCC risk among individuals seronegative for HBsAg and anti-HCV. These variants were linked to a higher long-term HCC risk, with individuals carrying multiple risk alleles showing notably elevated risk. These findings offer valuable insights into potential biomarkers for HCC risk stratification, and further investigation is warranted to elucidate the underlying mechanisms of hepatocarcinogenesis associated with these variants.

Affiliations

[1]Institute of Clinical Medicine, National Yang Ming Chiao Tung University, Taipei, Taiwan; [2]Division of Gastroenterology and Hepatology, Department of Internal Medicine, National Taiwan University Hospital, Taipei, Taiwan; [3]Hepatitis Research Center, National Taiwan University Hospital, Taipei, Taiwan; [4]Division of Urology, Department of Surgery, Chang Gung Memorial Hospital, Linkou, Taoyuan, Taiwan; [5]School of Medicine, Chang Gung University, Taoyuan, Taiwan; [6]Division of Gastroenterology and Hepatology, Department of Medicine, Taipei Veterans General Hospital, Taipei, Taiwan; [7]Division of Gastroenterology and Hepatology, Department of Internal Medicine, Taichung Veterans General Hospital, Taichung, Taiwan; [8]Department of Internal Medicine, Taichung Veterans General Hospital Chiayi Branch, Chiayi, Taiwan; [9]School of Medicine, National Yang Ming Chiao Tung University, Taipei, Taiwan; [10]Institute of Medicine, Chung Shan Medical University, Taichung, Taiwan; [11]Department of Medical Research, Chung Shan Medical University Hospital, Taichung, Taiwan; [12]Kaohsiung Municipal Siaogang Hospital, Kaohsiung, Taiwan; [13]College of Medicine, Kaohsiung Medical University, Kaohsiung, Taiwan; [14]Division of Endocrinology, Department of Internal Medicine, Koo Foundation Sun Yat-Sen Cancer Center, Taipei, Taiwan; [15]Department of Genomic Medicine and Center for Medical Genetics, Changhua Christian Hospital, Changhua, Taiwan; [16]Department of Obstetrics and Gynecology, Changhua Christian Hospital, Changhua, Taiwan;

[17]Department of Medical Research, Changhua Christian Hospital, Changhua, Taiwan; [18]Cardiovascular and Mitochondrial Related Disease Research Center, Hualien Tzu Chi Hospital, Buddhist Tzu Chi Medical Foundation, Hualien, Taiwan; [19]Graduate Institute of Medical Science, China Medical University, Taichung, Taiwan; [20]Department of Medical Research, China Medical University Hospital, China Medical University, Taichung, Taiwan; [21]Department of Medical Laboratory Science and Biotechnology, Asia University, Taichung, Taiwan; [22]Center of General Education, Buddhist Tzu Chi Medical Foundation, Tzu Chi University of Science and Technology, Hualien, Taiwan; [23]Institute of Molecular and Genomic Medicine, National Health Research Institute, Taiwan; [24]Institute of Statistical Science, Academia Sinica, Taipei, Taiwan; [25]Genomics Research Center, Academia Sinica, Taipei, Taiwan; [26]Hepatobiliary Division, Department of Internal Medicine, Kaohsiung Medical University Hospital, Kaohsiung, Taiwan; [27]Center of Excellence for Metabolic Associated Fatty Liver Disease, National Sun Yat-sen University, Kaohsiung, Taiwan; [28]Graduate Institute of Clinical Medicine, National Taiwan University College of Medicine, Taipei, Taiwan; [29]Advanced Therapeutics Research Center, National Yang Ming Chiao Tung University, Taipei, Taiwan

Abbreviations

AFP, alpha-fetoprotein; ALT, alanine aminotransferase; GWAS, genome-wide association study; HCC, hepatocellular carcinoma; HBV, hepatitis B virus; HCV, hepatitis C virus; MASLD, metabolic dysfunction-associated steatotic liver disease; OR, odds ratio; PCA, principal component analysis; PNPLA3, patatin-like phospholipase domain-containing protein 3; SAMM50, sorting and assembly machinery component 50 homolog; TLCN, Taiwan Liver Cancer Network.

Financial support

This study was supported by the National Science and Technology Council, Taipei, Taiwan (grant: 112-2628-B-A49-007 and 113-2628-B-A49-012), the Higher Education Sprout Project by the Ministry of Education (MOE) in Taiwan, and by the National Health Research Institute, Chunan, Taiwan (grant: NHRI-EX112-11117PI). None of the funding organizations contributed to the study design and delivery, data collection, management, analysis, and interpretation, data preparation and review, or manuscript approval.

Conflict of interest

The authors have no conflicts of interest to disclose.

Please refer to the accompanying ICMJE disclosure forms for further details.

Authors' contributions

Study concept and design: Mei-Hsuan Lee. Data acquisition: Mei-Hsuan Lee. Data analysis and interpretation: Tsai-Hsuan Yang, Xia-Rong Liu, Tung-Hung Su, Szu-Ching Yin, Fen-Fang Chen, and Mei-Hsuan Lee. Coordination and oversight of sample collection for the TPMI biobank: See-Tong Pang, Ming-Chih Hou, Yen-Chun Peng, Shun-Fa Yang, Peng-Ju Huang, Sing-Lian Lee, Ming Chen, and Chih-Yang Huang. Drafting of the manuscript: Tsai-Hsuan Yang, Xia-Rong Liu, Szu-Ching Yin, and Mei-Hsuan Lee. Critical revision of the manuscript for important intellectual content: Tsai-Hsuan Yang, Xia-Rong Liu, Tung-Hung Su, Szu-Ching Yin, Yi-Ting Chen, Fen-Fang Chen, See-Tong Pang, Ming-Chih Hou, Yen-Chun Peng, Shun-Fa Yang, Peng-Ju Huang, Sing-Lian Lee, Ming Chen, Chih-Yang Huang, Ya-Hsuan Chang, Hsuan-Yu Chen, Hwai-I Yang, Ming-Lung Yu, Chien-Jen Chen, Jia-Horng Kao, and Mei-Hsuan Lee. Funding acquisition and study supervision: Mei-Hsuan Lee.

Data availability

All or part of the data used in this research were authorized by and received from Taiwan Biobank (Authorization Code: TWBR11104-1) and Health and Welfare Data Science Center Database, Ministry of Health and Welfare (NHIRD_MOHW: H112006). Dr. Mei-Hsuan Lee applied for all data use. Other researchers may request the materials used through collaboration.

Acknowledgments

We gratefully acknowledge the National Health Research Institute Biobank and its cooperative institutions for providing biological specimens and related clinical data (all de-identified) for this research. The National Health Research Institute Biobank is supported by grants from the Ministry of Health and Welfare, the Ministry of Science and Technology, and the National Health Research Institutes, Taiwan. We also extend our gratitude to all participants and investigators of the Taiwan Precision Medicine Initiative. This study was funded by Academia Sinica (Grant Nos. 40-05-GMM, AS-GC-110-MD02, and 236e-1100202) and the National Development Fund, Executive Yuan (Grant No. NSTC 111-3114-Y-001-001).

Supplementary data

Supplementary data to this article can be found online at https://doi.org/10.1016/j.jhepr.2025.101673.

References

Author names in bold designate shared co-first authorship

[1] **Mak LY**, **Liu K**, **Chirapongsathorn S**, et al. Liver diseases and hepatocellular carcinoma in the Asia-Pacific region: burden, trends, challenges and future directions. Nat Rev Gastroenterol Hepatol 2024;21(12):834–851.

[2] Vogel A, Meyer T, Sapisochin G, et al. Hepatocellular carcinoma. Lancet 2022;400:1345–1362.

[3] Hassan MM, Spitz MR, Thomas MB, et al. The association of family history of liver cancer with hepatocellular carcinoma: a case-control study in the United States. J Hepatol 2009;50:334–341.

[4] Turati F, Edefonti V, Talamini R, et al. Family history of liver cancer and hepatocellular carcinoma. Hepatology 2012;55:1416–1425.

[5] Yang TH, Chan C, Yang PJ, et al. Genetic susceptibility to hepatocellular carcinoma in patients with chronic hepatitis virus infection. Viruses 2023;15.

[6] **Jiang DK**, **Sun J**, **Cao G**, et al. Genetic variants in STAT4 and HLA-DQ genes confer risk of hepatitis B virus-related hepatocellular carcinoma. Nat Genet 2013;45:72–75.

[7] Zhang H, Zhai Y, Hu Z, et al. Genome-wide association study identifies 1p36.22 as a new susceptibility locus for hepatocellular carcinoma in chronic hepatitis B virus carriers. Nat Genet 2010;42:755–758.

[8] Lee MH, Huang YH, Chen HY, et al. Human leukocyte antigen variants and risk of hepatocellular carcinoma modified by hepatitis C virus genotypes: a genome-wide association study. Hepatology 2018;67:651–661.

[9] Kumar V, Kato N, Urabe Y, et al. Genome-wide association study identifies a susceptibility locus for HCV-induced hepatocellular carcinoma. Nat Genet 2011;43:455–458.

[10] Matsuura K, Sawai H, Ikeo K, et al. Genome-wide association study identifies TLL1 variant associated with development of hepatocellular carcinoma after eradication of hepatitis C virus infection. Gastroenterology 2017;152:1383–1394.

[11] Miki D, Ochi H, Hayes CN, et al. Variation in the DEPDC5 locus is associated with progression to hepatocellular carcinoma in chronic hepatitis C virus carriers. Nat Genet 2011;43:797–800.

[12] Kim KY, Kim JO, Kim YS, et al. Genome-wide association of individual vulnerability with alcohol-associated liver disease: a Korean genome and epidemiology study. Hepatology 2022;75:391–402.

[13] **Trepo E**, **Caruso S**, **Yang J**, et al. Common genetic variation in alcohol-related hepatocellular carcinoma: a case-control genome-wide association study. Lancet Oncol 2022;23:161–171.

[14] Wang Z, Budhu AS, Shen Y, et al. Genetic susceptibility to hepatocellular carcinoma in chromosome 22q13.31, findings of a genome-wide association study. JGH Open 2021;5:1363–1372.

[15] Ishigaki K, Akiyama M, Kanai M, et al. Large-scale genome-wide association study in a Japanese population identifies novel susceptibility loci across different diseases. Nat Genet 2020;52:669–679.

[16] Hassan MM, Li D, Han Y, et al. Genome-wide association study identifies high-impact susceptibility loci for HCC in North America. Hepatology 2024;80:87–101.

[17] Huang SF, Chang IC, Hong CC, et al. Metabolic risk factors are associated with non-hepatitis B non-hepatitis C hepatocellular carcinoma in Taiwan, an endemic area of chronic hepatitis B. Hepatol Commun 2018;2:747–759.

[18] Lee MH, Yang HI, Jen CL, et al. Community and personal risk factors for hepatitis C virus infection: a survey of 23,820 residents in Taiwan in 1991-2. Gut 2011;60:688–694.

[19] Wu HC, Jeng WJ, Pan MH, et al. Incidence of hepatocellular carcinoma in a community-based Taiwanese population without chronic HBV/HCV infection. JHEP Rep 2022;4:100410.
[20] Ko CL, Lin WZ, Lee MT, et al. Genome-wide association study reveals ethnicity-specific SNPs associated with ankylosing spondylitis in the Taiwanese population. J Transl Med 2022;20:589.
[21] Chen YT, Chen TI, Yin SC, et al. Prevalence, proportions of elevated liver enzyme levels, and long-term cardiometabolic mortality of patients with metabolic dysfunction-associated steatotic liver disease. J Gastroenterol Hepatol 2024;39:1939–1949.
[22] Liu YL, Patman GL, Leathart JB, et al. Carriage of the PNPLA3 rs738409 C >G polymorphism confers an increased risk of non-alcoholic fatty liver disease associated hepatocellular carcinoma. J Hepatol 2014;61:75–81.
[23] **Vilar-Gomez E**, **Pirola CJ**, **Sookoian S**, et al. PNPLA3 rs738409 and risk of fibrosis in NAFLD: exploring mediation pathways through intermediate histological features. Hepatology 2022;76:1482–1494.
[24] Valenti L, Al-Serri A, Daly AK, et al. Homozygosity for the patatin-like phospholipase-3/adiponutrin I148M polymorphism influences liver fibrosis in patients with nonalcoholic fatty liver disease. Hepatology 2010;51:1209–1217.
[25] Ahmed A, Cule M, Bell JD, et al. Differing genetic variants associated with liver fat and their contrasting relationships with cardiovascular diseases and cancer. J Hepatol 2024;81(6):921–929.
[26] Romeo S, Huang-Doran I, Baroni MG, et al. Unravelling the pathogenesis of fatty liver disease: patatin-like phospholipase domain-containing 3 protein. Curr Opin Lipidol 2010;21:247–252.
[27] **Romeo S**, **Kozlitina J**, Xing C, et al. Genetic variation in PNPLA3 confers susceptibility to nonalcoholic fatty liver disease. Nat Genet 2008;40:1461–1465.
[28] Kitamoto T, Kitamoto A, Yoneda M, et al. Genome-wide scan revealed that polymorphisms in the PNPLA3, SAMM50, and PARVB genes are associated with development and progression of nonalcoholic fatty liver disease in Japan. Hum Genet 2013;132:783–792.
[29] Anstee QM, Darlay R, Cockell S, et al. Genome-wide association study of non-alcoholic fatty liver and steatohepatitis in a histologically characterised cohort(☆). J Hepatol 2020;73:505–515.
[30] **Chen Y**, **Du X**, Kuppa A, et al. Genome-wide association meta-analysis identifies 17 loci associated with nonalcoholic fatty liver disease. Nat Genet 2023;55:1640–1650.
[31] Smagris E, BasuRay S, Li J, et al. Pnpla3I148M knockin mice accumulate PNPLA3 on lipid droplets and develop hepatic steatosis. Hepatology 2015;61:108–118.
[32] Chen VL, Oliveri A, Miller MJ, et al. PNPLA3 genotype and diabetes identify patients with nonalcoholic fatty liver disease at high risk of incident cirrhosis. Gastroenterology 2023;164:966–977 e917.
[33] Trepo E, Nahon P, Bontempi G, et al. Association between the PNPLA3 (rs738409 C>G) variant and hepatocellular carcinoma: evidence from a meta-analysis of individual participant data. Hepatology 2014;59:2170–2177.
[34] Chalasani N, Vilar-Gomez E, Loomba R, et al. PNPLA3 rs738409, age, diabetes, sex, and advanced fibrosis jointly contribute to the risk of major adverse liver outcomes in metabolic dysfunction-associated steatotic liver disease. Hepatology 2024;80(5):1212–1226.
[35] **Grimaudo S, Pipitone RM**, Pennisi G, et al. Association between PNPLA3 rs738409 C>G variant and liver-related outcomes in patients with nonalcoholic fatty liver disease. Clin Gastroenterol Hepatol 2020;18:935–944 e933.
[36] Rosso C, Caviglia GP, Birolo G, et al. Impact of PNPLA3 rs738409 polymorphism on the development of liver-related events in patients with nonalcoholic fatty liver disease. Clin Gastroenterol Hepatol 2023;21:3314–3321 e3313.
[37] Chen L, Dong J, Liao S, et al. Loss of Sam50 in hepatocytes induces cardiolipin-dependent mitochondrial membrane remodeling to trigger mtDNA release and liver injury. Hepatology 2022;76:1389–1408.
[38] Sung JL. Hepatitis B virus infection and its sequelae in Taiwan. Gastroenterol Jpn 1984;19:363–366.
[39] Nischalke HD, Lutz P, Bartok E, et al. The PNPLA3 I148M variant promotes lipid-induced hepatocyte secretion of CXC chemokines establishing a tumorigenic milieu. J Mol Med (Berl) 2019 Nov;97(11):1589–1600.
[40] **Nischalke HD**, **Schmalz F**, Buch S, et al. Genetic variation of SAMM50 is not an independent risk factor for alcoholic hepatocellular carcinoma in caucasian patients. Int J Mol Sci 2022;23(23):15353.
[41] Pe'er I, Yelensky R, Altshuler D, et al. Estimation of the multiple testing burden for genomewide association studies of nearly all common variants. Genet Epidemiol 2008 May;32(4):381–385.
[42] **Sakaue S**, **Kanai M**, Tanigawa Y, et al. A cross-population atlas of genetic associations for 220 human phenotypes. Nat Genet 2021 Oct;53(10):1415–1424.

Keywords: long-term risk; prospective cohort; biobank; Steatosis; susceptibility.

Received 17 June 2025; received in revised form 30 October 2025; accepted 3 November 2025; Available online 11 November 2025

Journal of Hepatology, Volume *8*

Supplemental information

Genome-wide association study identifies three PNPLA3/SAMM50 SNPs associated with HCC development in non-viral liver disease

Xia-Rong Liu, Tsai-Hsuan Yang, Tung-Hung Su, Szu-Ching Yin, Yi-Ting Chen, Fen-Fang Chen, See-Tong Pang, Ming-Chih Hou, Yen-Chun Peng, Shun-Fa Yang, Peng-Ju Huang, Sing-Lian Lee, Ming Chen, Chih-Yang Huang, Ya-Hsuan Chang, Hsuan-Yu Chen, Hwai-I Yang, Ming-Lung Yu, Chien-Jen Chen, Jia-Horng Kao, and Mei-Hsuan Lee

Genome-wide association study identifies three PNPLA3/SAMM50 SNPs associated with HCC development in non-viral liver disease

Xia-Rong Liu, Tsai-Hsuan Yang, Tung-Hung Su, Szu-Ching Yin, Yi-Ting Chen, Fen-Fang Chen, See-Tong Pang, Ming-Chih Hou, Yen-Chun Peng, Shun-Fa Yang, Peng-Ju Huang, Sing-Lian Lee, Ming Chen, Chih-Yang Huang, Ya-Hsuan Chang, Hsuan-Yu Chen, Hwai-I Yang, Ming-Lung Yu, Chien-Jen Chen, Jia-Horng Kao, Mei-Hsuan Lee

Table of contents

Table S1……………………………………………………………………………..2

Table S2……………………………………………………………………………..4

Table S3……………………………………………………………………………..5

Table S4……………………………………………………………………………..6

Table S5……………………………………………………………………………..7

Table S6……………………………………………………………………………..8

Table S7……………………………………………………………………………..9

Table S8…………………………………………………………………………….10

Table S1. Baseline characteristics of the study population in the GWAS phase.

**Baseline characteristics**	**HCC cases** (n = 765) n	%	**non-HCC controls** (n = 9949) n	%	**P-value**
**Sex**					<.0001
Male	606	79.22	2827	28.41	
Female	159	20.78	7122	71.59	
**Age** (years)					<.0001
30-39	22	2.88	565	5.68	
40-49	49	6.41	1662	16.71	
50-59	152	19.87	3118	31.34	
60-69	217	28.37	3858	38.78	
≥70	325	42.48	746	7.5	
**BMI** (kg/m2)					<.0001
<18.5	31	4.16	277	2.79	
18.5-24	293	39.33	4927	49.56	
24-27	223	29.93	2761	27.77	
≥27	198	26.58	1976	19.88	
Missing	20		8		
**Serum ALT** (IU/L)					<.0001
<15	65	8.53	2721	27.35	
15-45	495	64.96	6554	65.88	
≥45	202	26.51	674	6.77	
Missing	3		0		<.0001
**Serum AFP** (ng/mL)					
<5	260	34.35	8881	89.27	
≥5	497	65.65	1068	10.73	
Missing	8		0		<.0001
**Alcohol consumption**					
Never	406	54.35	9043	90.9	
Ever	341	45.65	905	9.1	
Missing	18		1		
**Cigarette smoking**					<.0001
Never	350	46.85	8312	83.55	
Ever	397	53.14	1637	16.45	
Missing	18		0		
**Fatty liver†**					<.0001
No	623	81.44	5607	56.40	
Yes	142	18.56	4335	43.60	
Missing	0		7		

† Fatty liver: Diagnosed by abdominal ultrasonography.

# Abbreviations: HCC, hepatocellular carcinoma. BMI, body mass index. ALT, alanine aminotransferase. AFP, alpha-fetoprotein.

Table S2. Frequency distribution of the 10 HCC-associated SNPs in the GWAS discovery sets.

		**HCC** (n=765)		**non-HCC control** (n=9949)		
**SNP**	**A1A2**	n	%	n	%	**P-value**
PNPLA3						
**rs738409**	CC	240	31.37	3714	37.33	<.0001
	CG	347	45.36	4813	48.38	
	GG	178	23.27	1422	14.29	
rs738408	CC	241	31.50	3728	37.47	<.0001
	TC	347	45.36	4805	48.30	
	TT	177	23.14	1416	14.23	
rs3747207	GG	246	32.16	3790	38.09	<.0001
	GA	348	45.49	4780	48.05	
	AA	171	22.35	1379	13.86	
rs4823173	GG	242	31.63	3682	37.01	<.0001
	GA	345	45.10	4822	48.47	
	AA	178	23.27	1445	14.52	
**rs2281135**	GG	232	30.33	3608	36.26	<.0001
	GA	352	46.01	4846	48.71	
	AA	181	23.66	1495	15.03	
rs2896019	TT	231	30.20	3604	36.22	<.0001
	TG	354	46.27	4845	48.70	
	GG	180	23.53	1500	15.08	
rs2294915	CC	230	30.07	3595	36.13	<.0001
	TC	353	46.14	4845	48.70	
	TT	182	23.79	1509	15.17	
SAMM50						
**rs2235776**	CC	233	30.46	3652	36.71	<.0001
	TC	360	47.06	4850	48.75	
	TT	172	22.48	1447	14.54	
rs2143571	GG	231	30.20	3616	36.35	<.0001
	GA	359	46.93	4861	48.86	
	AA	175	22.88	1472	14.80	
rs2073080	CC	230	30.07	3614	36.33	<.0001
	TC	361	47.19	4864	48.89	
	TT	174	22.75	1471	14.79	

Abbreviations: HCC, hepatocellular carcinoma. A1A2, genotype.

Table S3. HCC risk by genotypes of the 3 HCC-associated SNPs in the GWAS discovery stratified by the presence of fatty liver.

		Without Fatty Liver† (5607 controls, 623 cases)		With Fatty Liver† (4335 controls, 142 cases)	
**SNP**	**Genotype**	**Crude OR (95% CI)**	**Multivariate adjusted OR¶ (95% CI)**	**Crude OR (95% CI)**	**Multivariate adjusted OR¶ (95% CI)**
**rs738409**	CC	1.00 (reference)	1.00 (reference)	1.00 (reference)	1.00 (reference)
	CG	1.17 (0.97-1.41)	1.12 (0.83-1.50)	1.07 (0.72-1.62)	1.12 (0.67-1.86)
	GG	2.21 (1.74-2.79)	2.32 (1.58-3.43)	2.13 (1.37-3.32)	2.10 (1.18-3.74)
		P_{trend} <.0001	P_{trend} =0.0002	P_{trend} =0.0016	P_{trend} =0.0157
**rs2281135**	GG	1.00 (reference)	1.00 (reference)	1.00 (reference)	1.00 (reference)
	GA	1.17 (0.97-1.42)	1.16 (0.86-1.55)	1.12 (0.74-1.70)	1.25 (0.74-2.09)
	AA	2.11 (1.67-2.67)	2.04 (1.38-3.01)	2.14 (1.37-3.35)	2.31 (1.29-4.13)
		P_{trend} <.0001	P_{trend} =0.0012	P_{trend} =0.0013	P_{trend} =0.0058
**rs2235776**	CC	1.00 (reference)	1.00 (reference)	1.00 (reference)	1.00 (reference)
	TC	1.21 (1.00-1.46)	1.28 (0.95-1.72)	1.15 (0.77-1.72)	1.12 (0.68-1.86)
	TT	2.18 (1.72-2.76)	2.16 (1.46-3.20)	1.83 (1.16-2.88)	1.71 (0.95-3.10)
		P_{trend} <.0001	P_{trend} =0.0003	P_{trend} =0.0136	P_{trend} =0.0887

† Fatty liver: Diagnosed by abdominal ultrasonography.

¶ Adjusted for the first 20 principal components, age, sex, BMI, cigarette smoking, alcohol consumption, serum ALT levels, and serum AFP levels.

# Abbreviations: HCC, hepatocellular carcinoma. A1A2, genotype. OR, odds ratio. CI, confidence interval.

Table S4. Odds ratios of the association of the three SNPs with HCC among cirrhotic patients.

SNP	GWAS discovery phase (152 HCC cases, 52 non-HCC controls) Crude OR (95%)	GWAS discovery phase Multi-adjusted* OR (95%)	Validation phase Hospital-based case-control study (104 HCC cases, 138 non-HCC controls) Crude OR (95%)	Validation phase Multi-adjusted§ OR (95%)
**rs738409**				
CC	1.00 (reference)	1.00 (reference)	1.00 (reference)	1.00 (reference)
GC	4.33 (2.07-9.08)	6.02 (1.79-20.24)	1.46 (0.79-2.72)	1.45 (0.76-2.77)
GG	10.13 (3.81-26.9)	12.39 (2.80-54.87)	3.22 (1.63-6.47)	3.06 (1.52-6.31)
P trend	<.0001	0.0006	<.0001	0.0020
**rs2281135**				
GG	1.00 (reference)	1.00 (reference)	1.00 (reference)	1.00 (reference)
AG	4.27 (2.04-8.95)	5.51 (1.67-16.31)	1.51 (0.82-2.83)	1.44 (0.76-2.78)
AA	10.31 (3.88-27.39)	10.99 (2.56-47.27)	3.06 (1.53-6.27)	2.93 (1.42-6.15)
P trend	<.0001	0.0008	0.0020	0.0040
**rs2235776**				
CC	1.00 (reference)	1.00 (reference)	1.00 (reference)	1.00 (reference)
TC	3.80 (1.87-7.71)	6.40 (1.95-20.94)	1.58 (0.86-2.93)	1.49 (0.80-2.81)
TT	18.53 (5.21-65.91)	25.49 (4.34-149.54)	2.31 (1.14-4.75)	2.22 (1.06-4.71)
P trend	<.0001	0.0001	0.0200	0.0350

*Adjusted for sex, age, BMI, smoking, alcohol, ALT, and AFP.
§ Adjusted for sex, age, BMI, ALT, and diabetes.
# Abbreviations: HCC, hepatocellular carcinoma. OR, odds ratio. CI, confidence interval.

Table S5. Baseline characteristics of the study population in the validation sets.

Baseline characteristics	Community-based set (N=855)				Hospital-based set (N=5930)			
	**HCC** (n=171)		**non-HCC control** (n=684)		**HCC** (n=470)		**non-HCC control** (n=5460)	
	n	%	n	%	n	%	n	%
**Sex**								
Male	103	60.2	412	60.2	298	63.4	2533	46.4
Female	68	39.8	272	39.8	172	36.6	2927	53.6
**Age** (years)								
30-39	16	9.4	63	9.2	17	3.6	507	9.3
40-49	38	22.2	151	22.1	36	7.7	864	15.8
50-59	71	41.5	286	41.8	102	21.7	1239	22.7
60-69	46	26.9	184	26.9	140	29.8	1547	28.3
≥70	0		0		175	37.2	1303	37.2
**BMI** (kg/m^2)								
<18.5	10	5.8	18	2.6	24	5.1	224	4.1
18.5-24	59	34.5	315	46.1	175	37.2	2128	39.0
24-27	50	29.2	217	31.7	129	27.4	1481	27.1
≥27	52	30.4	134	19.6	142	30.2	1627	29.8
**Serum ALT** (IU/L)								
<15	98	57.3	463	67.7	67	14.3	1071	19.6
15-45	61	35.7	203	29.7	290	61.7	3275	60.0
≥45	12	7.0	18	2.6	113	24.0	1114	20.4
**Alcohol consumption**								
Never	137	80.1	615	90.0				
Ever	34	19.9	68	10.0				
Missing	0		1					
**Cigarette smoking**								
Never	102	59.6	470	68.7				
Ever	69	40.4	214	31.3				

Abbreviations: HCC, hepatocellular carcinoma. BMI, body mass index. ALT, alanine aminotransferase.

Table S6. HCC risk by genotypes of the 3 HCC-associated SNPs in the GWAS validation stratified by the BMI.

SNP	Genotype	**Hospital-based set** (470 cases, 5460 controls)			
		BMI < 24 $^{kg}/_{m^2}$ (199 cases, 2352 controls)		BMI ≥ 24 $^{kg}/_{m^2}$ (271 cases, 3108 controls)	
		**Crude OR (95% CI)**	**Multivariate adjusted OR¶ (95% CI)**	**Crude OR (95% CI)**	**Multivariate adjusted OR¶ (95% CI)**
**rs738409**	CC	1.00 (reference)	1.00 (reference)	1.00 (reference)	1.00 (reference)
	GC	1.23 (0.88, 1,73)	1.25 (0.89, 1.76)	0.95 (0.71, 1.28)	0.98 (0.73, 1.32)
	GG	1.82 (1.20, 2.73)	1.73 (1.13, 2.62)	1.89 (1.37, 2.63)	1.93 (1.38, 2.69)
		P_{trend} =0.006	P_{trend} =0.012	P_{trend} <0.001	P_{trend} <0.001
**rs2281135**	GG	1.00 (reference)	1.00 (reference)	1.00 (reference)	1.00 (reference)
	AG	1.21 (0.86, 1.69)	1.21 (0.86, 1.71)	0.94 (0.70, 1.27)	0.96 (0.71, 1.30)
	AA	1.62 (1.07, 2.43)	1.54 (1.01, 2.33)	1.82 (1.32, 2.53)	1.87 (0.34, 2.60)
		P_{trend} =0.024	P_{trend} =0.047	P_{trend} <0.001	P_{trend} <0.001
**rs2235776**	CC	1.00 (reference)	1.00 (reference)	1.00 (reference)	1.00 (reference)
	TC	1.27 (0.91, 1.78)	1.28 (0.92, 1.81)	1.05 (0.79, 1.42)	1.06 (0.79, 1.43)
	TT	1.46 (0.95, 2.21)	1.39 (0.89, 2.13)	1.70 (1.22, 2.38)	1.75 (1.24, 2.45)
		P_{trend} =0.065	P_{trend} =0.105	P_{trend} =0.004	P_{trend} =0.003

¶ Adjusted for sex, age, serum ALT levels, and diabetes.

# Abbreviations: HCC, hepatocellular carcinoma. BMI, body mass index. OR, odds ratio. CI, confidence interval.

Table S7. Baseline characteristics of the study population in the prospective cohort.

Baseline characteristics	Total (N=67909)		HCC (n=32)		non-HCC control (n=67877)		
	n	%	n	%	n	%	P-value
**Sex**							<.0001
Male	22514	33.2	11	34.4	22503	33.2	
Female	45395	66.8	21	65.6	45374	66.9	
**Age** (years)							<.0001
30-39	17271	25.4	0	0	17271	25.4	
40-49	16419	24.2	3	9.4	16416	24.2	
50-59	18738	27.6	8	25.0	18730	27.6	
60-69	15183	22.4	20	62.5	15163	22.3	
≥70	298	0.4	1	3.1	297	0.4	
**BMI** (kg/m^2)							0.0021
<18.5	2429	3.6	0	0	2429	3.6	
18.5-24	33123	48.8	6	18.8	33117	48.8	
24-27	17988	26.5	15	46.9	17973	26.5	
≥27	14369	21.2	11	34.4	14358	21.2	
**Serum ALT** (IU/L)							<.0001
<15	21913	32.3	5	15.6	21908	32.3	
15-45	40649	59.9	18	56.3	40631	59.9	
≥45	5344	7.9	9	28.1	5335	7.9	
Missing	3		0		3		
**Alcohol consumption**							<.0001
Never	62170	91.6	22	68.8	62148	91.6	
Ever	5693	8.4	10	31.3	5683	8.4	
Missing	46		0		46		
**Cigarette smoking**							0.0018
Never	54978	81.0	19	59.4	54959	81.0	
Ever	12904	19.0	13	40.6	12891	19.0	
Missing	27		0		27		

Abbreviations: HCC, hepatocellular carcinoma. BMI, body mass index. ALT, alanine aminotransferase.

Table S8. Validation of the HLA signal from GWAS results in the prospective cohort.

	**Total** (2n=135818)		**Control** (2n=135754)		**HCC** (2n=64)		
HLA-DPB1	2n	%	2n	%	2n	%	**P-value**
DPB1*02:01	21486	15.8	21476	15.8	10	15.6	0.9659
DPB1*02:02	10074	7.4	10070	7.4	4	6.3	0.1887
DPB1*04:01	10987	8.1	10984	8.1	3	4.7	0.1285
DPB1*05:01	59547	43.8	59513	43.8	34	53.1	0.1344
DPB1*13:01	8644	6.4	8638	6.4	6	9.4	0.3237
Rare (freq<5% mix)	25080	18.5	25073	18.5	7	10.9	0.1205
